# GIVING BACK TO FEEL GOOD? SOCIAL RESPONSIBILITY, AGE, AND NEGATIVE AFFECT

**DOI:** 10.1093/geroni/igac059.2397

**Published:** 2022-12-20

**Authors:** Madeline Nichols, Dakota Witzel, Robert Stawski

**Affiliations:** Oregon State University, Corvallis, Oregon, United States; Penn State University, State College, Pennsylvania, United States; Oregon State University, Corvallis, Oregon, United States

## Abstract

Developmental theories suggest that midlife and older adulthood are stages in which individuals may begin to focus their time on contributing to society (Erikson, 1969). During these stages, individuals may engage in socially responsible behaviors that protect against negative affect resulting from a lower sense of purpose in later life (Greenfield & Marks, 2004). Social responsibility includes both subjective measures of an individual’s felt contribution to society (i.e., generativity) and objective measures reporting actual time volunteering in different settings (Rossi, 2001). We utilized data from the Midlife in the United States Refresher survey study and Biomarker Project (N=735, Mage=51.56, SD=13.59, 50.20% Male) to explore how self-perceptions of generativity and time spent volunteering predicted negative affect for individuals in midlife and older adulthood. Preliminary analyses indicate that higher generativity (p<.001) and older age (p<.001), but not average time spent volunteering, were associated with higher negative affect. Further, we considered age as a potential moderator for the associations between generativity, volunteering, and negative affect. Age significantly interacted with generativity (p<.01), such that the effects of generativity on reducing negative affect decrease with age. Age did not significantly interact with time spent volunteering. Discussion will focus on how actual engagement in socially responsible behaviors and perceived societal contributions might yield different outcomes regarding protection against negative affect in mid- and later-life. Future directions may include exploring daily indicators of time spent volunteering, generative beliefs, and affect.

